# Association between Serum Concentrations of Persistent Organic Pollutants and Self-Reported Cardiovascular Disease Prevalence: Results from the National Health and Nutrition Examination Survey, 1999–2002

**DOI:** 10.1289/ehp.10184

**Published:** 2007-05-25

**Authors:** Myung-Hwa Ha, Duk-Hee Lee, David R. Jacobs

**Affiliations:** 1 Department of Preventive Medicine and Health Promotion Research Center, School of Medicine, Kyungpook National University, Daegu, Korea; 2 National Cancer Center, Ilsan, Korea; 3 Division of Epidemiology, School of Public Health, University of Minnesota, Minnesota, USA; 4 Department of Nutrition, University of Oslo, Oslo, Norway

**Keywords:** cardiovascular diseases, dioxin, persistent organic pollutants, pesticides, polychlorinated biphenyls

## Abstract

**Background:**

There is now increasing evidence that exposure to persistent organic pollutants (POPs) can contribute to the development of inflammatory diseases such as atherosclerosis.

**Objective:**

The objective of this study was to examine associations of serum concentrations of POPs with self-reported history of cardiovascular disease (CVD).

**Design:**

Cross-sectional associations of serum POPs concentrations with the prevalence of self-reported CVD were investigated in 889 adults ≥ 40 years of age in the National Health and Nutrition Examination Survey, 1999–2002. We selected 21 POPs [3 polychlorinated dibenzo-*p*-dioxins (PCDDs), 3 polychlorinated dibenzofurans (PCDFs), 5 dioxin-like polychlorinated biphenyls (PCBs), 6 nondioxin-like PCBs, and 4 organochlorine (OC) pesticides] because they were detectable in ≥ 60% of participants.

**Results:**

Dioxin-like PCBs, nondioxin-like PCBs, and OC pesticides were positively associated with the prevalence of CVD only among females. Compared with those in the lowest quartile of serum concentration, the odds ratios for CVD across increasing quartiles were 0.9, 2.0, and 5.0 for dioxin-like PCBs (*p* for trend < 0.01), 1.2, 1.2, and 3.8 for nondioxin-like PCBs (*p* for trend < 0.01), and 1.9, 1.7, and 4.0 for OC pesticides (*p* for trend = 0.03). PCDDs showed positive trends with the prevalence of CVD in both males and females; adjusted odds ratios were 1.4, 1.7, and 1.9 (*p* for trend = 0.07, males and females combined).

**Conclusions:**

Our findings need to be carefully interpreted because of the cross-sectional design and use of self-reported CVD. Prospective studies are needed to clarify these associations.

Persistent organic pollutants (POPs) are a family of lipophilic stable chemicals that bio-accumulate in adipose tissue and create a lasting toxic body burden (Van den Berg et al. 2006). In addition to various known deleterious effects, POPs have recently been implicated as a possible cause of cardiovascular disease (CVD) (Mastin 2005). The association between POPs and CVD is plausibly causal through several biological mechanisms. Exposure to polychlorinated biphenyls (PCBs) or 2,3,7,8-tetrachlorodibenzo-*p*-dioxin (TCDD) is known to increase atherogenic serum lipid levels in both animals and humans (Bombick et al. 1984; Lovati et al. 1984; Swift et al. 1981). In addition, these contaminants cause direct damage to endothelial cells via oxidative stress (Hennig et al. 2002; Stegeman et al. 1995; Toborek et al. 1995). The combination of an elevation in serum lipids with damage to endothelial cells would be expected to increase the risk of CVD. There is also epidemiologic evidence for the association of POPs with CVD, although it is not totally consistent. Weakly or modestly elevated rate ratios for mortality from ischemic heart disease have been found in several cohorts, in which there was occupational or accidental relatively brief exposure to high doses of several POPs (Bertazzi et al. 1998, 2001; Calvert et al. 1998; Gustavsson and Hogstedt 1997; Hooiveld et al. 1998; Pesatori et al. 1998, 2003; Steenland et al. 1999; Vena et al. 1998). One recent study reported that residents living in ZIP code areas contaminated with POPs had a statistically significant elevation in both coronary heart disease (CHD) and acute myocardial infarction hospital discharge rates compared with clean ZIP codes (Sergeev and Carpenter 2005).

There is ongoing controversy concerning health effects of background, enduring environmental exposure to endocrine disruptors such as POPs (Kaiser 2000). There is a need for epidemiologic study in the general population because extrapolation from studies of high exposure to selected POPs in occupational or accidental settings may not be appropriate to background exposure (Lee et al. 2006a). In support of this assertion, we recently reported a striking dose–response relation between serum concentrations of POPs and diabetes in the general population with background exposure to POPs (Lee et al. 2006b).

Serum concentrations of biologically important POPs or their metabolites, including dioxins and furans [polychlorinated dibenzo-*p*-dioxins (PCDDs) and polychlorinated dibenzofurans (PCDFs)], PCBs, hexachloro-benzene (HCB), and several organochlorines (OCs) used as pesticides, were measured in subsamples of the National Health and Nutrition Examination Survey (NHANES), 1999–2000 and 2001–2002 [National Center for Health Statistics (NCHS) 2005]. The present study was performed to investigate associations of prevalent self-reported CVD with the serum concentrations of POPs; we restricted the study to POPs that were widely prevalent in order to ensure a valid CVD prevalence estimate in the referent group with low exposure.

## Methods

The 1999–2000 and 2001–2002 NHANES conducted by the Centers for Disease Control and Prevention (CDC) were designed to be nationally representative of the noninstitutionalized, U.S. civilian population on the basis of a complex, multistage probability sample. Approximately 9,965 persons, 2 months to 85 years of age, were studied in NHANES 1999–2000, and 11,039 persons were included in NHANES 2001–2002. Details of the NHANES protocol and all testing procedures are available elsewhere (NCHS 2006a, 2006b). The study protocol was reviewed and approved by the CDC institutional review board; additionally, informed written consent was obtained from all subjects before they took part in the study.

PCDDs, PCDFs, PCBs, and OC pesticides were measured in serum from a random one-third subsample of people ≥ 12 years of age in 1999 and 2000. In 2001 and 2002, PCDDs, PCDFs, and coplanar PCBs were measured in a random one-third subsample of people ≥ 20 years of age, and OC pesticides and other PCBs were measured in these people and in a random one-third subsample of people ≥ 12–19 years of age.

The NHANES data collection included a standardized home interview followed by a detailed physical examination in a mobile evaluation clinic or the participant’s home. Information on demographic characteristics, ethnicity, and medical history of diabetes was obtained in a household interview. Venous blood and urine samples were collected and shipped weekly at –20°C. PCDDs, PCDFs, PCBs, and OC pesticides were all measured as individual chemicals by high-resolution gas chromatography/high-resolution mass spectrometry using isotope dilution for quantification. All of these analytes were measured in approximately 5 mL serum using a modification of the method of Turner et al. (1997). The POPs were reported on a lipid-adjusted basis using concentrations of serum total cholesterol and triglycerides.

Although 49 POPs were measured in both NHANES 1999–2000 and 2001–2002, to avoid bias in estimation among those below the limit of detection (LOD), we selected the 21 POPs for which at least 60% of study subjects had concentrations > LOD: 3 PCDDs, 3 PCDFs, 5 dioxin-like PCBs, 6 nondioxin-like PCBs, and 4 OC pesticides. There were a total of 1,054 study participants ≥ 40 years of age with information on serum concentrations of the 21 selected POPs. After excluding 165 diabetic participants, including newly diagnosed cases, the final sample size was 889. We excluded those with diabetes from the present study because simple adjustment for diabetic status may not be enough to exclude its effect due to strong associations between serum concentrations of POPs and diabetes (Lee et al. 2006b). However, inclusion of diabetic patients did not materially change results.

For each POP, subjects with serum concentrations < LOD were regarded as the reference group, and subjects with detectable values were categorized by cutoff points of 25th, 50th, and 75th percentile values. To yield a cumulative measure of three PCDDs, we summed the ranks according to magnitude of detectable levels of the three POPs that belong to the PCDDs, using the rank 0 for any nondetectable value. The summary values were categorized by cutoff points of 25th, 50th, and 75th percentile values. We assigned and cumulated POP subclasses similarly for the three PCDFs, the five dioxin-like PCBs, the six nondioxin-like PCBs, and the four OC pesticides. Thus, depending on the sum of ranks of the several POPs belonging to the specific POP subclass under consideration, the subject could be in the lowest quartile or in a higher quartile; however, if all POPs in the subclass were nondetectable, the subject would be placed in the lowest quartile.

Participants were considered to have prevalent CVD if they answered “yes” to any of the following questions:

- “Has a doctor or other health professional ever told you that you had CHD?”
- “Has a doctor or other health professional ever told you that you had angina/angina pectoris?”
- “Has a doctor or other health professional ever told you that you had heart attack/myocardial infarction?”
- “Has a doctor or other health professional ever told you that you had a stroke?”

We used logistic regression models to calculate multivariate-adjusted odds ratios (ORs). Adjusting CVD risk factors were age (years), race/ethnicity, poverty income ratio (continuous), body mass index (BMI; continuous), cigarette smoking (never, former, or current), cotinine levels (nanograms per milligram), alcohol consumption (grams per day), leisure time physical activity (vigorous, moderate, or none), status of hypertension (yes/no), total cholesterol (continuous), HDL (high-density lipoprotein)-cholesterol (continuous), triglyceride (continuous), and C-reactive protein (continuous). We substituted median values of noncases for missing BMI, poverty income ratio, cotinine levels, or alcohol consumption in 152 subjects. Exclusion of these individuals did not change any conclusions, but it did limit power in some analyses; therefore, they were retained.

All statistical analyses were performed using SAS 9.1 (SAS Institute Inc., Cary, NC, USA) and SUDAAN 9.0 (Research Triangle Institute, Research Triangle Park, NC, USA). Estimates of main results were calculated accounting for stratification and clustering (Korn and Graubard 1991), and adjusting for age, race, ethnicity, and poverty income ratio instead of using sample weights; this adjustment is regarded as a good compromise between efficiency and bias (Graubard and Korn 1999; Korn and Graubard 1991). Because results were very similar with both SAS 9.1 and SUDAAN 9.0, we present the results based on SAS 9.1.

## Results

The sample of 889 participants included 48% males and 55% whites. Mean age ± SD was 60.4 ± 13.6 years (range, 40–85 years). In Table 1, we present associations of known CVD risk factors with five subclasses of POPs, rather than the 21 specific POPs, because our final conclusion was made based on the results of these five subclasses. Age was the strongest and most important correlate of serum concentrations of all five subclasses of POPs in both sexes. However, associations of other CVD risk factors with POPs appeared to be substantially different depending on specific classes of POPs. Subjects with white race had lower concentrations of OC pesticides in both sexes and of PCDDs in females, but higher PCDFs or PCBs especially among females. Those with greater income had higher concentrations of PCBs but lower OC pesticides. Obese people tended toward higher concentrations of most POPs, except nondioxin-like PCBs among females. Smokers tended to have lower concentrations of some POPs, whereas drinkers had higher concentrations of PCBs. HDL cholesterol, total cholesterol, and triglycerides were variously associated depending on subclasses of POPs or sex. HDL cholesterol was positively associated with PCBs in both sexes, but inversely associated with OC pesticides in females. Because the serum concentrations of POPs used in this study were already adjusted for both total cholesterol and triglyceride, most POPs were not associated or were even inversely associated with total cholesterol or triglycerides, except positive associations between OC pesticides with triglycerides. When lipid-unadjusted POPs levels were used, all five subclasses of POPs were positively associated with both total cholesterol and triglycerides (data not shown). C-reactive protein was positively associated with OC pesticides in both sexes, but was inversely associated with nondioxin-like PCBs among females. After adjusting for age, we found positive pairwise correlations among serum concentrations of the five subclasses of POPs with correlation coefficients of 0.32–0.84 in males and 0.28–0.86 in females.

We found 108 prevalent self-reported CVD cases (61 males and 47 females). They consisted of 87 CHD cases (sum of CHD, angina, and heart attack) and 40 stroke cases; some persons reported more than one condition. Table 2 shows associations between the five subclasses of POPs and the prevalence of CVD by sex. PCDDs showed positive trends with the prevalence of CVD in both males and females, even though analyses stratified by sex failed to reach statistical significance. When both sexes were combined, the adjusted ORs were 1.4, 1.7, and 1.9 (*p* for trend = 0.07). PCDFs were unassociated with the prevalence of CVD in either sex. Dioxin-like PCBs, nondioxin-like PCBs, and OC pesticides showed significantly positive associations with the prevalence of CVD only among females. Adjusted ORs across quartiles of each subclass were 0.9, 2.0, and 5.0 (*p* for trend < 0.01); 1.2, 1.2, and 3.8 (*p* for trend < 0.01); and 1.9, 1.7, and 4.0 (*p* for trend = 0.03) for dioxin-like PCBs, nondioxin-like PCBs, and OC pesticides, respectively. In the fully adjusted models, serum levels of HDL cholesterol, total cholesterol, and triglycerides were included to eliminate residual confounding, even though lipid adjusted POPs concentrations were used. However, dropping individual lipids from the list of covariates did not change results.

In Tables 3 and 4, we further examined associations of prevalence of CVD with specific POPs belonging to subclasses that showed positive associations in Table 2. In the case of PCDDs, we presented the results in both males and females (Table 3). Among the three PCDDs, only 1,2,3,6,7,8-hexachlorodibenzo-*p*-dioxin showed significant trends in both males and females. Even though 1,2,3,4,6,7,8-heptachlorodibenzo-*p*-dioxin and 1,2,3,4,6,7,8,9-octachlorodibenzo-*p*-dioxin did not show linear trends, the risk appeared to substantially increase from the first or second category of detectable range compared with nondetectable value, especially in males. On the other hand, most dioxin-like PCBs, nondioxin-like PCBs, and OC pesticides showed dose–response relations with CVD in females (Table 4).

In all analyses, we further considered self-reported weight loss in the past 1 or 10 years as possible confounders because weight loss increases serum concentrations of POPs and because patients with a history of CVD may intentionally decrease their body weight after diagnosis. However, the adjustment for weight loss did not change the results (data not shown). When the dataset was reanalyzed using the 87 CHD cases as the outcome variable, the trends were very similar with those of CVD cases (data not shown).

## Discussion

This cross-sectional study demonstrated that the background exposure to POPs was positively associated with the prevalence of CVD in the U.S. general population. Details of associations were substantially different depending on specific subclasses of POPs or sex, but the associations with CVD suggested by the data are as strong as those of traditional CVD risk factors.

Our findings are in general agreement with, but stronger than, those of previous prospective cohort studies among subjects exposed to high concentrations of selected POPs in occupational or accidental settings (Bertazzi et al. 1998, 2001; Calvert et al. 1998; Gustavsson and Hogstedt 1997; Hooiveld et al. 1998; Pesatori et al. 1998, 2003; Steenland et al, 1999; Vena et al. 1998). Considering that exposure levels in the present study were much lower than those in previous studies, this may be a puzzling finding. Interestingly, we also reported striking dose–response relations between serum concentrations of POPs and prevalent diabetes in the same NHANES dataset (Lee et al. 2006b). In a recent editorial (Lee et al. 2006a), we discussed that epidemiologic studies in general populations who are neither occupationally nor accidentally exposed may be critical to investigate possible health effects of POPs in humans. Specifically, previous studies often failed to select a true reference group with very low exposure levels to POPs, for example, by examining people with very low serum concentrations of POPs, despite persistence of the POPs in adipose tissue and consistent background environmental exposure to them. They generally did not examine the possibility that a mixture of POPs could have an additive or synergistic effect on health, as we have done by employing a summary measure that considered joint health effects of POPs with possibly different toxicologic properties. These studies did not consider the possibility of nonlinear associations (Lee et al. 2006a). All of these points can lead to substantial underestimation of risk or masking of true risk.

On the other hand, the fact that CVD was associated with various POPs with different toxicologic profiles may also be consistent with the possibility that the observed relations are not causal. It is entirely possible that the POPs investigated in the present study are not themselves causally related to CVD. Rather, they could be surrogates of exposure to a mixture of POPs in the general population because there are high correlations among serum concentrations of various POPs in the human body. Furthermore, we can not completely exclude the possibility of reverse causality; CVDs may alter metabolism so as to increase serum concentrations of POPs. However, if reverse causality were occurring, it would be sensible to expect that all POPs in both sexes would be associated with CVD, rather than selected POPs as we observed.

The NHANES data provided a unique chance to investigate the possible associations between serum concentrations of various POPs and CVD in a random sample of the general population, despite the recognition that self-reported CVD as the dependent variable is less accurate than physician-diagnosed CVD. Previous epidemiologic studies have focused on selected populations, often occupationally or accidentally exposed to high levels of selected POPs.

In the present study, the specific subclasses of POPs related to CVD appeared to differ by sex. Serum concentrations of PCDDs were positively associated with prevalence of CVD among males, but females showed strong positive associations with dioxin-like or non-dioxin-like PCBs or OC pesticides. It is well-known that males and females differ in many aspects of vulnerability to environmental xenobiotics and other stressors (Gochfeld 2006). Differences between the sexes in the response of nonreproductive cells, in addition to reproductive cells, to TCDD or PCBs have been observed in several animal studies (Enan et al. 1996; Vega-Lopez et al. 2007; Wyde et al. 2001). Because most occupational epidemiologic studies on POPs have been performed among men, it is largely unknown whether various health effects of POPs between men and women are similar or not. In our previous study of diabetes (Lee et al. 2006b), the associations of POPs did not differ between sexes.

There is increasing experimental evidence that exposure to POPs such as TCDD or PCBs can lead to cardiovascular toxicity and atherosclerosis. A number of biochemical changes induced by POPs observed in *in vitro* or *in vivo* experimental studies are viewed as atherogenic. PCBs or TCDD can compromise the normal function of vascular endothelial cells by activating oxidative stress–sensitive signaling pathways and subsequent proinflammatory events critical in the pathology of atherosclerosis and CVD (Hennig et al. 2002; Stegeman et al. 1995; Toborek et al. 1995). In addition, exposure to TCDD increased serum cholesterol, triglyceride, and phospholipids and suppressed low-density lipoprotein receptors in the liver (Bombick et al. 1984; Lovati et al. 1984; Swift et al. 1981). Moreover, TCDD promoted the differentiation of macrophages to atherogenic foam cells or deregulated several genes in cell proliferation and apoptosis in smooth muscle cell (Dalton et al. 2001; Vogel et al. 2004).

Unlike evidence from experimental studies in which the affinity to aryl hydrocarbon receptor (AhR) was important to induce atherosclerosis (Hennig et al. 2002; Stegeman et al. 1995; Toborek et al. 1995), the strengths of association of each POP belonging to the category of PCDDs or PCDFs did not appear to be correlated with the toxic equivalent factors (TEFs) of each POP. The concept of TEFs, a measure of the ability to bind to the AhR, was developed to facilitate risk assessment and regulatory control of exposure to complex PCDD, PCDF, and PCB mixtures (Van den Berg et al. 2006). Also, in the present study, nondioxin-like PCBs appeared to show more consistent and stronger associations than dioxin-like PCBs. Even among the dioxin-like PCBs, PCBs with low TEFs tended to show stronger associations than those with high TEFs. Our previous study of the associations between POPs and diabetes similarly reported no relation between strength of association and TEF of each POP (Lee et al. 2006b). These findings suggest that the affinity to AhR may not be a critical pathway of toxicity of POPs in humans for some outcomes, unlike findings from cells or animal models. Alternatively, the associations of some POPs with CVD observed in the present study may not be direct as we discussed above.

The present study has several limitations, primarily because of its cross-sectional design, but also because diagnosis of CVD was self-reported and fatal events were not even considered. The expense and blood volume needed to measure POPs in a population sample are such that such data are rare; therefore, the NHANES data may offer important insights, despite these limitations. In the case of misdiagnosis, we expect that the misclassification would be nondifferential, leading to the underestimation of ORs. Misclassification bias is also possible because some subjects with a higher POP value but a lower sample volume could be classified in the reference group, or vice versa. Such misclassification is also likely to be nondifferential because sample volume is probably unrelated to prevalence of CVD.

In summary, we found positive associations between serum concentrations of some POPs and the prevalence of CVD in this sample of the U.S. population. Thus, prospective study of the relation between background dioxin exposure and validated CVD should be a priority in further study of these associations. Both the exposure and the disease have substantial prevalence, and the public health significance of a causal relation of POPs with CVD should be noted.

## Figures and Tables

**Table 1:** Age-adjusted Spearman correlation coefficients^a^ between five categories of lipid-adjusted POPs with demographic or cardiovascular risk factors by sex.

	PCDDs	PCDFs	Dioxin-like PCBs	Nondioxin-like PCBs	OC pesticides
Males					
Age	0.39**	0.23**	0.48**	0.44**	0.47**
Race	NS	NS	NS	NS	–0.20**
Poverty income ratio	NS	NS	0.14**	NS	NS
BMI	0.21**	0.10*	0.10*	NS	0.20**
Current smoker	–0.16**	NS	–0.12*	NS	NS
Exercise	NS	NS	NS	NS	–0.11*
Alcohol consumption	NS	NS	0.11*	0.10*	NS
HDL cholesterol	NS	NS	NS	0.14**	NS
Total cholesterol	NS	NS	NS	NS	NS
Triglycerides	NS	NS	NS	NS	0.18**
C-reactive protein	NS	NS	NS	NS	0.11*
Females					
Age	0.42**	0.36**	0.62**	0.51**	0.57**
Race	NS	0.10*	0.10*	NS	–0.30**
Poverty income ratio	NS	NS	0.10*	0.10*	–0.18**
BMI	0.10*	NS	NS	–0.14**	0.19**
Current smoker	–0.25**	–0.10*	NS	NS	NS
Exercise	NS	NS	NS	NS	NS
Alcohol consumption	NS	NS	NS	NS	NS
HDL cholesterol	NS	NS	0.10*	0.12**	–0.17**
Total cholesterol	NS	–0.15**	–0.09*	NS	NS
Triglycerides	NS	–0.10*	NS	–0.13**	0.16**
C-reactive protein	NS	NS	NS	–0.10*	0.15**

NS, not significant. For race, white = 1 and others = 0. For current smoker, current = 1 and others = 0. For exercise, yes = 1 and no = 0.

a Before calculating correlation coefficients, detectable values of each POP were individually ranked, and the rank order of the individual POPs in each subclass were summed to arrive at the subclass value; all nondetectable values were ranked as 0.

* *p* < 0.05.

** *p* < 0.01.

**Table 2:** Number of cases/total number and adjusted OR (95% CI) for prevalence of cardiovascular diseases by quartiles of PCDDs, PCDFs, dioxin-like PCBs, nondioxin-like PCBs, and OC pesticides in males and females.

Analyte	< 25th	25th to < 50th	50th to < 75th	≥ 75th	*p*_trend_
Males					
PCDDs	7/106	12/107	19/107	23/107	
	Referent	1.7 (0.6–4.7)	2.1 (0.8–5.9)	2.2 (0.8–6.1)	0.14
PCDFs	13/106	12/107	17/107	19/107	
	Referent	0.7 (0.3–1.8)	0.9 (0.4–2.2)	0.7 (0.3–1.7)	0.60
Dioxin-like PCBs	6/106	16/107	17/107	22/107	
	Referent	2.2 (0.8–6.5)	1.8 (0.6–5.3)	1.7 (0.6–5.5)	0.64
Nondioxin-like PCBs	7/106	17/107	14/107	23/107	
	Referent	2.3 (0.8–6.4)	1.3 (0.5–3.9)	1.8 (0.6–5.0)	0.61
OC pesticides	10/106	12/107	18/108	21/106	
	Referent	0.7 (0.2–1.9)	0.9 (0.3–2.4)	0.9 (0.3–2.3)	0.96
Females					
PCDDs	8/115	9/116	11/116	19/115	
	Referent	1.1 (0.3–3.3)	1.5 (0.5–4.3)	2.0 (0.7–6.4)	0.16
PCDFs	9/115	10/116	13/116	15/115	
	Referent	0.9 (0.3–2.5)	1.1 (0.4–3.0)	1.0 (0.3–2.8)	0.92
Dioxin-like PCBs	4/115	8/116	12/116	23/115	
	Referent	0.9 (0.2–3.5)	2.0 (0.5–7.6)	5.0 (1.2–20.4)	< 0.01
Nondioxin-like PCBs	5/115	9/115	9/117	24/115	
	Referent	1.2 (0.4–4.0)	1.2 (0.4–4.2)	3.8 (1.1–12.8)	0.02
OC pesticides	3/115	9/116	10/116	25/115	
	Referent	1.9 (0.5–7.7)	1.7 (0.4–7.1)	4.0 (1.0–17.1)	0.03

ORs were adjusted for age, race, poverty income ratio, BMI, cigarette smoking, serum cotinine, alcohol consumption, exercise, HDL cholesterol, total cholesterol, triglycerides, hypertension, and C-reactive protein. Detectable values of each POP were individually ranked, and the rank orders of the individual POPs in each subclass were summed to arrive at the subclass value. All not detectable values were ranked as 0. The summary values were categorized by cutoff points of 25th, 50th, and 75th values of the sum of ranks.

**Table 3:** Concentration (pg/g of lipid), number of cases/total number, and adjusted OR (95% CI) for prevalence of self-reported cardiovascular diseases by category of PCDDs in males and females.

			Detectable				
Analyte	Detection rate (%)	Not detectable	< 25th	25th to < 50th	50th to < 75th	≥ 75th	*p*_trend_
Males							
1,2,3,6,7,8-Hexachlorodibenzo-*p*-dioxin	82.2	—	26.6^a^	42.1	61.3	111	
		5/76	3/86	14/89	21/89	18/87	
		Referent	0.9 (0.2–4.0)	4.3 (1.3–14.2)	4.1 (1.3–12.6)	2.5 (0.8–7.7)	0.04
1,2,3,4,6,7,8-Heptachlorodibenzo-*p*-dioxin	89.5	—	20.7	40.0	61.7	111	
		3/45	12/95	17/96	11/96	18/95	
		Referent	3.2 (0.8–13.3)	3.7 (0.9–15.4)	1.7 (0.4–7.6)	2.4 (0.5–10.3)	0.94
1,2,3,4,6,7,8,9-Octachlorodibenzo-*p*-dioxin	82.0	—	192	310	469	899	
		4/77	7/87	18/88	16/88	16/87	
		Referent	1.5 (0.4–6.0)	3.7 (1.1–12.6)	2.7 (0.8–9.6)	2.1 (0.6–7.7)	0.28
Females							
1,2,3,6,7,8-Hexachlorodibenzo-*p*-dioxin	83.8	—	27.6	45.9	66.1	111	
		6/75	5/96	3/97	12/97	21/97	
		Referent	1.0 (0.3–3.7)	0.4 (0.1–1.9)	1.7 (0.5–5.2)	2.8 (0.9–8.6)	0.04
1,2,3,4,6,7,8-Heptachlorodibenzo-*p*-dioxin	93.1	—	25.5	50.2	76.6	135	
		3/32	12/107	7/108	11/108	14/107	
		Referent	1.6 (0.3–7.9)	1.3 (0.2–7.7)	2.2 (0.4–12.0)	1.9 (0.3–10.8)	0.45
1,2,3,4,6,7,8,9-Octachlorodibenzo-*p*-dioxin	92.9	—	278	445	660	1,170	
		4/33	10/107	4/107	12/108	17/107	
		Referent	0.6 (0.1–2.4)	0.2 (0.1–1.1)	0.7 (0.2–2.7)	0.7 (0.2–2.8)	0.74

ORs were adjusted for age, race, poverty income ratio, BMI, cigarette smoking, serum cotinine, alcohol consumption, exercise, HDL cholesterol, total cholesterol, triglycerides, hypertension, and C-reactive protein.

a Median concentrations are displayed in each category.

**Table 4:** Concentration (ng/g of lipid), number of cases/total number, and adjusted OR (95% CI) for prevalence of cardiovascular diseases by categories of specific POPs belonging to dioxin-like PCBs, nondioxin-like PCBs, and OC pesticides in females.

			Detectable				
Analyte	Detection rate (%)	Not detectable	< 25th	25th to < 50th	50th to < 75th	≥ 75th	*p*_trend_
Dioxin-like PCBs							
2,4,4′,5-Tetrachlorobiphenyl (PCB-74)	87.2	—	8.2^a^	13.9	20.5	36.1	
		2/59	6/100	8/101	8/101	23/101	
		Referent	1.1 (0.2–6.1)	1.2 (0.2–6.4)	1.3 (0.2–7.3)	4.5 (0.8–24.8)	0.01
2,3′,4,4′,5-Pentachlorobiphenyl (PCB-118)	87.7	—	8.6	15.2	26.4	49.3	
		2/57	9/98	5/105	8/101	23/101	
		Referent	1.8 (0.3–9.4)	0.6 (0.1–4.1)	1.3 (0.2–7.8)	4.5 (0.8–25.5)	0.02
3,3′,4,4′,5-Pentachlorobiphenyl (PCB-126)	88.1	—	18.3	31.6	51.0	95.4	
		3/55	9/101	7/102	11/102	17/102	
		Referent	1.3 (0.3–5.8)	1.4 (0.3–6.6)	1.6 (0.4–7.4)	2.6 (0.6–12.2)	0.17
2,3,3′,4,4′,5-Hexachlorobiphenyl (PCB-156)	71.4	—	5.9	9.0	12.1	20.9	
		3/132	5/83	7/83	15/81	17/83	
		Referent	2.0 (0.4–9.5)	2.6 (0.6–12.0)	9.2 (2.1–39.4)	10.4 (2.3–46.7)	< 0.01
3,3′,4,4′,5,5′-Hexachlorobiphenyl (PCB-169)	89.6	—	13.3	21.7	32.4	51.0	
		3/48	5/103	8/104	16/103	15/104	
		Referent	0.6 (0.1–2.7)	0.9 (0.2–3.9)	1.2 (0.3–5.3)	1.2 (0.3–5.1)	0.38
Nondioxin-like PCBs							
2,2′,4,4′5-Pentachlorobiphenyl (PCB-99)	74.2	—	6.0	9.7	14.5	26.9	
		10/119	4/84	3/87	12/86	18/86	
		Referent	0.3 (0.1–1.1)	0.2 (0.1–1.0)	1.1 (0.4–3.0)	1.5 (0.5–3.9)	0.08
2,2′,3,4,4′,5-Hexachlorobiphenyl (PCB-138)	87.7	—	18.3	32.1	51.3	91.0	
		1/57	10/100	4/102	7/102	25/101	
		Referent	6.8 (0.8–58.9)	1.6 (0.2–15.9)	3.6 (0.4–32.8)	13.4 (1.6–115.0)	< 0.01
2,2′,4,4′,5,5′-Hexachlorobiphenyl (PCB-153)	90.3	—	27.3	48.5	71.8	127.0	
		1/45	7/104	8/105	9/103	22/105	
		Referent	3.7 (0.4–34.8)	3.0 (0.3–27.1)	3.6 (0.4–33.5)	10.4 (1.1–94.1)	< 0.01
2,2′,3,3′,4,4′,5-Heptachlorobiphenyl (PCB-170)	88.3	—	9.2	15.2	21.9	36.4	
		1/54	5/102	11/101	10/103	20/102	
		Referent	2.5 (0.3–23.3)	3.8 (0.4–33.3)	3.5 (0.4–32.1)	9.2 (1.0–84.5)	0.01
2,2′,3,4,4′,5,5′-Heptachlorobiphenyl (PCB-180)	92.2	—	18.9	34.6	51.3	86.4	
		1/36	6/107	10/106	10/107	20/106	
		Referent	1.8 (0.2–16.5)	2.0 (0.2–17.9)	2.0 (0.2–18.7)	4.5 (0.5–40.9)	0.07
2,2′,3,4′,5,5′,6-Heptachlorobiphenyl (PCB-187)	82.5	—	7.4	11.2	16.8	30.4	
		1/86	8/93	9/98	12/95	17/95	
		Referent	5.0 (0.6–43.5)	3.5 (0.4–30.2)	5.8 (0.7–50.1)	7.4 (0.9–63.6)	0.07
OC pesticides							
*p,p*′-Dichlorodiphenyltrichloroethane	100	—	189	556	1,145	2,440	
		—	8/115	9/116	10/116	20/115	
			Referent	0.8 (0.3–2.9)	0.7 (0.2–2.1)	1.7 (0.6–4.9)	0.22
Oxychlordane	93.7	—	12.1	20.2	31.4	50.5	
		0/29	3/108	9/108	9/110	26/107	
			Referent	1.9 (0.4–7.7)	1.7 (0.4–7.6)	6.8 (1.6–29.3)	< 0.01
*trans*-Nonachlor	98.3	—	15.4	27.2	42.4	80.8	
		0/8	2/113	10/114	12/114	23/113	
			Referent	3.7 (0.7–18.6)	3.4 (0.7–17.1)	6.5 (1.3–33.6)	0.03
Heptachlor epoxide	68.2	—	5.9	9.2	13.1	23.9	
		9/147	1/78	8/80	13/79	16/78	
		Referent	0.1 (0.1–1.3)	1.1 (0.3–3.2)	2.0 (0.7–5.8)	1.9 (0.6–5.7)	0.05

ORs were adjusted for age, race, poverty income ratio, BMI, cigarette smoking, serum cotinine, alcohol consumption, exercise, HDL cholesterol, total cholesterol, triglycerides, hypertension, and C-reactive protein.

a Median concentrations are displayed in each category.
